# Metabolic syndrome and pharmacotherapy outcomes in patients with type 2 diabetes mellitus

**DOI:** 10.3389/fcdhc.2024.1380244

**Published:** 2024-05-23

**Authors:** Shawqi H. Alawdi, Mohammed Al-Dholae, Salah Al-Shawky

**Affiliations:** ^1^ Department of Pharmacology, Faculty of Pharmacy, Syrian Private University (SPU), Damascus, Syria; ^2^ Department of Pharmacology, Faculty of Medicine, Thamar University, Dhamar, Yemen; ^3^ Department of Medicine, Faculty of Medicine, Thamar University, Dhamar, Yemen

**Keywords:** metabolic syndrome, pharmacotherapy outcomes, glycemic control, NCEP, cardiovascular diseases, hypertension

## Abstract

**Background:**

Metabolic syndrome is a group of metabolic abnormalities that increase predisposition to several diseases including ischemic heart disease and diabetes mellitus. The study aimed to investigate metabolic syndrome among patients with type-2 diabetes mellitus (DM), and its impact on pharmacotherapy outcomes.

**Methods:**

An observational cross-sectional study was performed on 910 patients with type-2 DM between June and December 2023. Fasting blood sugar, triglycerides, high-density lipoproteins (HDL), blood pressure, and abdominal obesity were measured. Metabolic syndrome was identified according to the National Cholesterol Education Program Adult Treatment Panel III criteria. Pharmacotherapy outcomes were assessed according to American Association of Clinical Endocrinologists and American Diabetes Association guidelines using the ability to achieve adequate glycemic control and normal levels of blood pressure and fasting plasma lipoproteins.

**Results:**

In total, 87.5% of type-2 DM patients had metabolic syndrome; the prevalence increased with age and was higher among females. Metabolic syndrome showed the following distribution of risk factors: insulin resistance (100%), low HDL (95.3%), elevated blood pressure (83%), triglycerides dyslipidemia (80.1%), and abdominal obesity (62.5%). Majority of the patients had either 5 or 4 risk factors of metabolic syndrome. The most common comorbidities were dyslipidemia (97.7%) and hypertension (83%). Treatment outcomes were insufficient where adequate glycemic control was only achieved in 12% of type-2 DM patients, and proper management of comorbid dyslipidemia and hypertension was achieved in 29% and 40.9% of patients, respectively. Adequate blood pressure control was less achieved in patients with metabolic syndrome (34.4%) than those without metabolic syndrome (77.2%). Similarly, dyslipidemia was less controlled in patients with metabolic syndrome (26.9%) than in those without metabolic syndrome (47.3%).

**Conclusion:**

Pharmacotherapy outcomes were inadequate for most patients with type-2 diabetes mellitus. Adopting early preventive and therapeutic interventions for metabolic syndrome is advised to improve treatment outcomes of the comorbid dyslipidemia and hypertension.

## Introduction

Metabolic syndrome represents an important global health problem brought about by the significant changes in our modern lifestyle. It is a group metabolic abnormalities that increase predisposition to several diseases including type 2 diabetes mellitus, cardiovascular diseases, and neurological disorders (1). These metabolic abnormalities include insulin resistance, abdominal obesity, high blood pressure, hypertriglyceridemia, and high density lipoprotein (HDL) dyslipidemia (2). Several criteria have been employed to diagnose metabolic syndrome, including the US National Cholesterol Educational Program Adult Treatment Panel III (NCEP ATP III) criteria, the International Diabetes Foundation (IDF) criteria, and the WHO criteria.

The significance of metabolic syndrome as a prognostic predictor for future negative health outcomes has been confirmed by several studies. For example, cardiovascular mortality was shown to be markedly increased among 4,483 patients with metabolic syndrome in Sweden and Finland (3). Additionally, the risk of death from ischemic cardiovascular disease was increased by 2.5 to 2.8-fold in patients with metabolic syndrome (4). Moreover, a meta-analysis of 87 studies using NCEP criteria to define metabolic syndrome confirmed a two-fold increase in cardiovascular diseases among patients with metabolic syndrome (5).

Early detection of risk factors for type 2 diabetes mellitus is an essential step to prevent diabetes mellitus type 2 as well as to decrease the likelihood of subsequent complications. Metabolic syndrome represents an ideal predictor of type 2 diabetes mellitus (6). Therefore, substantial consideration should be given to ameliorate metabolic syndrome as an interventional approach for the prevention of type 2 diabetes mellitus. The present study aimed to investigate the prevalence and clinical patterns of metabolic syndrome among a sample of type 2 diabetes mellitus patients in Yemen and the relevance of metabolic syndrome to the efficacy of anti-diabetic therapy.

## Methods

### Study design

The present study was a cross-sectional observational study conducted on a sample of 910 patients with type 2 diabetes mellitus recruited from two major tertiary hospitals in Sana, Yemen (Thawra and UST hospitals), during the period between June 2023 and December 2023.

### Participant selection

All patients with type 2 diabetes mellitus who attended to the outpatient clinics for follow-up during the study period and accepted to participate in the study were enrolled. The inclusion criteria were patients with type 2 diabetes mellitus aged > 18 years old and have been on regular pharmacotherapy for type 2 diabetes mellitus for at least six months. The exclusion criteria were pregnant women and newly diagnosed type 2 diabetes mellitus patients of less than six months.

### Study variables and measurements

Body weight was obtained and expressed in kilograms (kg), and height was measured and expressed in centimeters (cm). Body mass index was then calculated by dividing weight in kilograms by height squared in meters. Waist circumference was measured around the abdomen at the level of the iliac crest. Blood pressure was measured using sphygmomanometer with an appropriate cuff size in sitting position by nurses after 10 minutes rest; both systolic and diastolic blood pressures were recorded. These data were taken by physical examination of the enrolled patients at hospitals during their ordinary follow-up. Fasting blood glucose, glycosylated hemoglobin (HbA1c), triglycerides, high density lipoproteins (HDL), low density lipoproteins (LDL), and total cholesterol levels were obtained from patient files and charts. Other data were collected using an interview questionnaire.

### Study endpoints

The study endpoints included achieving adequate glycemic control and blood pressure control. Adequate glycemic control was achieved when the fasting blood glucose was ≤ 126 mg/dl or HbA1c was ≤ 6.5%. Controlled levels of blood pressure were achieved when systolic blood pressure was ≤ 130 mmHg or diastolic blood pressure was ≤ 80 mmHg.

Metabolic syndrome was diagnosed according to criteria of the NCEP ATP III (7). The presence of three or more of the following five risk factors warrants metabolic syndrome diagnosis: waist circumference ≥ 88 cm in women and ≥ 102 cm in men to confirm abdominal obesity; elevated blood pressure (≥ 130/85 mmHg) or receiving treatment for hypertension; fasting blood glucose ≥ 100 mg/dl or receiving treatment for diabetes; elevated triglycerides ≥150 mg/dl or receiving treatment for elevated triglycerides; and low HDL ≤ 50 mg/dl in women and ≤ 40 mg/dl in men or receiving treatment for low HDL levels. Patients were classified according to their body mass index (BMI) into underweight (BMI less than 18.5), ideal weight (BMI between 18.5 and 24.9), overweight (BMI between 25 and 29.9), obese (BMI between 30 and 34.9), and severely obese (BMI more than 35).

### Ethical consideration

Ethical approval was obtained from the Institutional Review Board of Wahda University Hospital, Faculty of Medicine, Thamar University. After explaining objectives and details of the study, informed consent was taken from each respondent who accepted to participate in the study. Data were collected anonymously to ensure privacy, and confidentiality was kept.

### Statistical analysis

Statistical analysis was performed using Statistical Package for Social Science software (SPSS, version 26) and Microsoft Office Excel 2010 was used for data processing. The chi-squared test was used for the assessment of association between the nominal variables studied. A *p*-value of less than 0.05 was considered statistically significant.

## Results

### Metabolic syndrome

Out of total 910 patients with type 2 diabetes, metabolic syndrome was found in 796 (87.5%) patients. The study sample included 51.9% male and 48.1% female patients. The prevalence of metabolic syndrome was found to be higher in female (91.3%) than in male patients (83.9%), and increased with increasing age from 71.2% in patients aged under 35 years to 96.3% in patients over 75 years old (**Table 1**). Additionally, majority of the patients (89.1%) aged between 35 and 75 years, and only 3% were above 75 years and 8% were under 35 years old. Moreover, results showed 36.6% of the patients had an ideal body weight while 38.8% were overweight and 15.5% were obese. Only 3.4% of the patients were underweight and 5.7% were severely obese. About 16.5% of the patients were current smokers. Metabolic syndrome was highly prevalent among type 2 diabetes patients especially in women. These findings could be explained by late diagnosis of metabolic syndrome and adopting no lifestyle or therapeutic interventions to treat its risk factors.

**Table 1:** Demographic characteristics of the participant type 2 diabetes mellitus patients (n=910).

	Total patients n (%)	Metabolic syndrome n (%)		*p*-value
		Not established	Established	
**Metabolic syndrome**	910 (100)	114 (12.5)	796 (87.5)	0.000
Gender				
Male	472 (51.9)	76 (16.1)	396 (83.9)	0.002
Female	438 (48.1)	38 (8.7)	400 (91.3)	
Age categories				
<35 yrs	73 (8)	21 (28.8)	52 (71.2)	0.003
35–54 yrs	430 (47.3)	49 (11.4)	381 (88.6)	
55–75 yrs	380 (41.8)	43 (11.3)	337 (88.7)	
>75 yrs	27 (3)	1 (3.7)	26 (96.3)	
Body mass index				
<18.5 = underweight	31 (3.4)	13 (41.9)	18 (58.1)	0.000
18.5–24.9 = ideal weight	333 (36.6)	48 (14.4)	285 (85.6)	
25–29.9 = overweight	353 (38.8)	36 (10.2)	317 (89.8)	
30–34.9 = obese	141 (15.5)	9 (6.4)	132 (93.6)	
>35 = severely obese	52 (5.7)	5 (9.6)	47 90.4)	
Smoking				
Yes	150 (16.5)	26 (17.3)	124 (82.7)	0.041
No	760 (83.5)	85 (11.2)	675 (88.8)	

**Figure 1:**
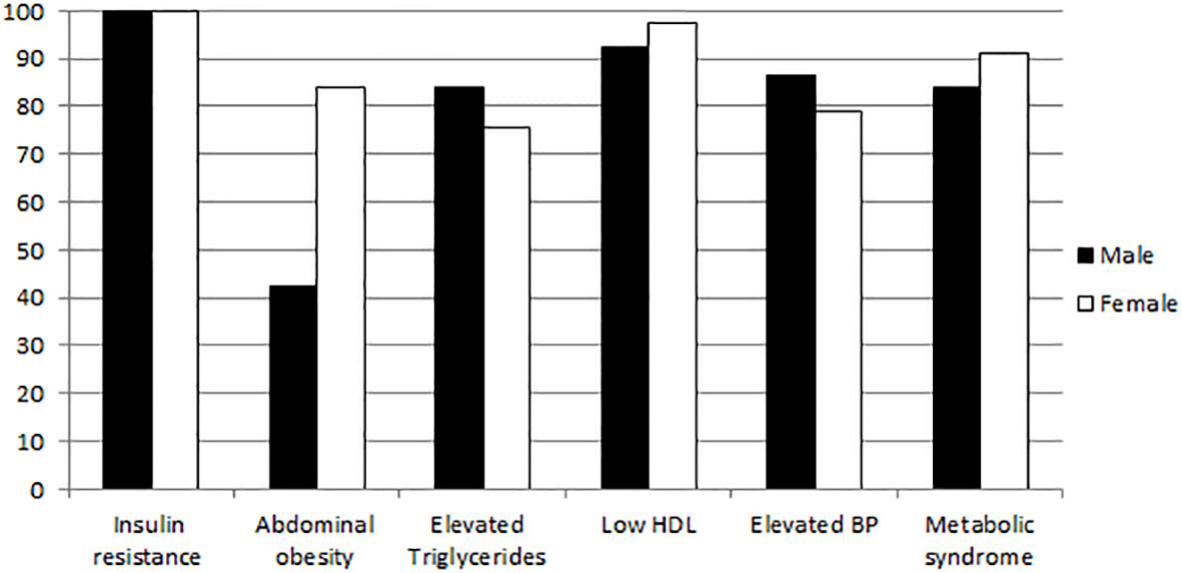
Prevalence of metabolic syndrome and its risk factors among type 2 diabetes mellitus patients (n=910).

The clinical patterns of metabolic syndrome in patients were showed as 3 risk factors in 24 (2.6%) patients, 4 risk factors in 338 (37.2%) patients, and 5 risk factors in 434 (47.7%) patients. The rest of the patients had at least 2 risk factors (12.5%). Components of metabolic syndrome were presented in **Table 2** and **Figure 1**. First, all patients had increased insulin resistance as they have type 2 diabetes mellitus. Additionally, abdominal obesity was found in 569 (63.4%) of the patients. Abdominal obesity was more prevalent among women (84%) than men (42.6%). Moreover, low HDL levels were observed in 867 (94.3%) of type 2 diabetes mellitus patients. Low HDL cholesterol levels were also more prevalent in women (97.3%) than men (92.2%). Furthermore, results showed that triglycerides levels were high in 729 (80.1%) of type 2 diabetes mellitus patients. In contrast, higher levels of serum triglycerides were observed among men (84.3%) than women (76.3%). Finally, the results showed high blood pressure in 755 (83%) of patients. Higher levels of blood pressure were also observed among men (86.6%) than women (79.0%). Higher prevalence of metabolic syndrome in patients with type 2 diabetes could be explained by the pervasive insulin resistance, HDL and triglyceride dyslipidemia, abdominal obesity, and elevated blood pressure. These factors interplay in vicious cycle with diabetes and cardiovascular, neurological, inflammatory, and endocrine complications.

**Table 2:** Components of metabolic syndrome in type 2 diabetes mellitus patients (n=910).

Risk factor	Total patients n (%)	Metabolic syndrome n (%)		*p*-value
		Not established	Established	
Insulin resistance				
FBG <100 mg/dL, not using hypoglycemic drugs	0 (0)	0 (0)	0 (0)	NA
FBG >100 mg/dL, or using hypoglycemic drugs	910 (100)	53 (5.8)	857 (94.2)	
Abdominal obesity				
Waist circumference male < 102 cm; female < 88 cm	341 (37.5)	106 (31.1)	235 (68.9)	0.000
Waist circumference male > 102 cm; female > 88 cm	569 (62.5)	5 (0.9)	564 (99.1)	
TGs dyslipidemia				
TGs<150 mg/dL; not using hypolipemic drugs	181 (19.9)	100 (55.2)	81 (44.8)	0.000
TGs>150 mg/dL; or using hypolipemic drugs	729 (80.1)	11 (1.5)	718 (98.5)	
HDL dyslipidemia				
HDL<40 mg/dL in male or <50 mg/dL in female; not using hypolipemic drugs	867 (95.3)	74 (8.5)	793 (91.5)	0.000
HDL>40 mg/dL in male or >50 mg/dL in female; using hypolipemic drugs	43 (4.7)	37 (86)	6 (14)	
Blood pressure				
SBP<130 mmHg, DBP<85 mmHg, or not using antihypertensive drugs	155 (17)	89 (57.4)	66 (42.6)	0.000
SBP>130 mmHg, DBP>85 mmHg, or using antihypertensive drugs	755 (83)	22 (2.9)	733 (97.1)	
Number of risk factors for metabolic syndrome				
Patients having 2 risk factors	114 (12.5)	114 (100)	0 (0)	0.000
Patients having 3 risk factors	24 (2.6)	0	24 (100)	
Patients having 4 risk factors	338 (37.2)	0	338 (100)	
Patients having 5 risk factors	434 (47.2)	0	434 (100)	

FBG, fasting blood glucose; TG, triglyceride; HDL, high-density lipoprotein; SBP, systolic blood pressure; DBP, diastolic blood pressure.

Almost all biochemical measurements of the enrolled patients were more aberrant in patients with metabolic syndrome than those without metabolic syndrome. These included fasting blood sugar, random blood sugar, glycosylated hemoglobin (HbA1c), triglycerides, high density lipoproteins (HDL), low density lipoproteins (LDL), and total cholesterol. Similarly, systolic and diastolic blood pressure as well as waist circumference were averaged higher in patients with metabolic syndrome than in patients without metabolic syndrome (**Table 3**). These biochemical and physical aberrations could be consequences for the underlying diminished lipoprotein lipase activity and increased HDL catabolism associated with insulin resistance.

**Table 3:** Physical and biochemical measurements of type2 diabetes mellitus patients (n=910).

Parameter	Metabolic syndrome (Mean ± SD)	
	Not established	Established
Systolic blood pressure	121.4 ± 20.5	138.2 ± 20.7
Diastolic blood pressure	76.8 ± 13.5	82.7 ± 12.5
Waist circumference (cm)	92.17 ± 15.7	100.6 ± 16.1
FBS (mg/dl)	158 ± 67.9	183.1 ± 74.2
RBS (mg/dl)	183.4 ± 66	236.8 ± 97.5
HbA1c (%)	9.2 ± 3	9.3 ± 2.5
Triglycerides (mg/dl)	161 ± 90.1	178 ± 93.7
HDL (mg/dl)	38.2 ± 12.9	35.3 ± 10.5
LDL (mg/dl)	109.5 ± 45.7	120.4 ± 51.4
Total cholesterol (mg/dl)	168.5 ± 63	184.5 ± 69

FBS, Fasting blood sugar; HbA1c, Glycosylate hemoglobin; HDL, High density lipoproteins; LDL, Low density lipoproteins; RBS, Random blood sugar; SD, Standard deviation.

### Pharmacotherapy assessment

The efficacy of anti-diabetic medications was evaluated by assessment the glycemic control. All patients included in the present study had been diagnosed with type 2 diabetes mellitus and were regularly taking their prescribed hypoglycemic drugs at least for six months prior to the beginning of the study. Nonetheless, adequate glycemic control was only achieved in 109 (12%) patients while 801 (88%) of the patients had inadequate glycemic control. Additionally, 839 (92.2%) patients had one or more comorbid cardiovascular diseases, namely hypertension in 755 (83%), ischemic heart diseases in 259 (28.5%), and congestive heart failure in 157 (17.3%) patients. Of these patients, 673 (80.2%) patients were treated with medications for their comorbid cardiovascular diseases. Adequate blood pressure control was achieved only in 275 (40.9%) patients. Moreover, 889 (97.7%) patients had comorbid dyslipidemia, and only 542 (61%) of these patients were treated with anti-dyslipidemia medications. Adequate plasma lipoprotein levels were only achieved in 157 (29%) of those treated patients. The inadequate glycemic control in most patients with type 2 diabetes necessitates the need to review pharmacotherapy and the patient compliance to prevent and treat the cardiovascular other complications associated with type 2 diabetes.

Further analysis of pharmacotherapy outcomes showed that low percentage of adequate glycemic control was achieved in both patients without metabolic syndrome (6.3%) and those with metabolic syndrome (12.8%). In the other hand, adequate blood pressure control was better achieved in patients without metabolic syndrome (77.2%) as compared with patients with metabolic syndrome (34.4%). In a similar vein, dyslipidemia was properly treated in patients without metabolic syndrome (47.3%) better than those with metabolic syndrome (26.9%). More details are represented in **Table 4**. Inadequate therapy of type2 diabetes mellitus, dyslipidemia, and hypertension could be attributed to several factors discussed below including insufficient prescribed pharmacotherapy, poor patient compliance, and untreated underlying risk factors of metabolic syndrome. Patient counseling could also be useful to reinforce pharmacotherapy outcomes.

**Table 4:** Pharmacotherapy outcomes of type 2 diabetes mellitus and its major comorbidities (n=910).

Disease	n (%)	Medication use n (%)	Metabolic syndrome	n (%)	Adequate control n (%)	Inadequate control n (%)	*p*-value
Diabetes mellitus	910 (100)	910 (100)	Yes	801 (88)	102 (12.8)	697 (87.2)	0.000
			No	109 (12)	7 (6.3%)	104 (93.7)	
Hypertension	755 (83)	673 (89.1)	Yes	572 (85)	197 (34.4)	375 (65.6)	0.000
			No	101 (15)	78 (77.2)	23 (22.8)	
Dyslipidemia	889 (97.7)	542 (61)	Yes	487 (89.9)	131 (26.9)	356 (73.1)	0.000
			No	55 (10.1)	26 (47.3)	29 (52.7)	

**Figure 2:**
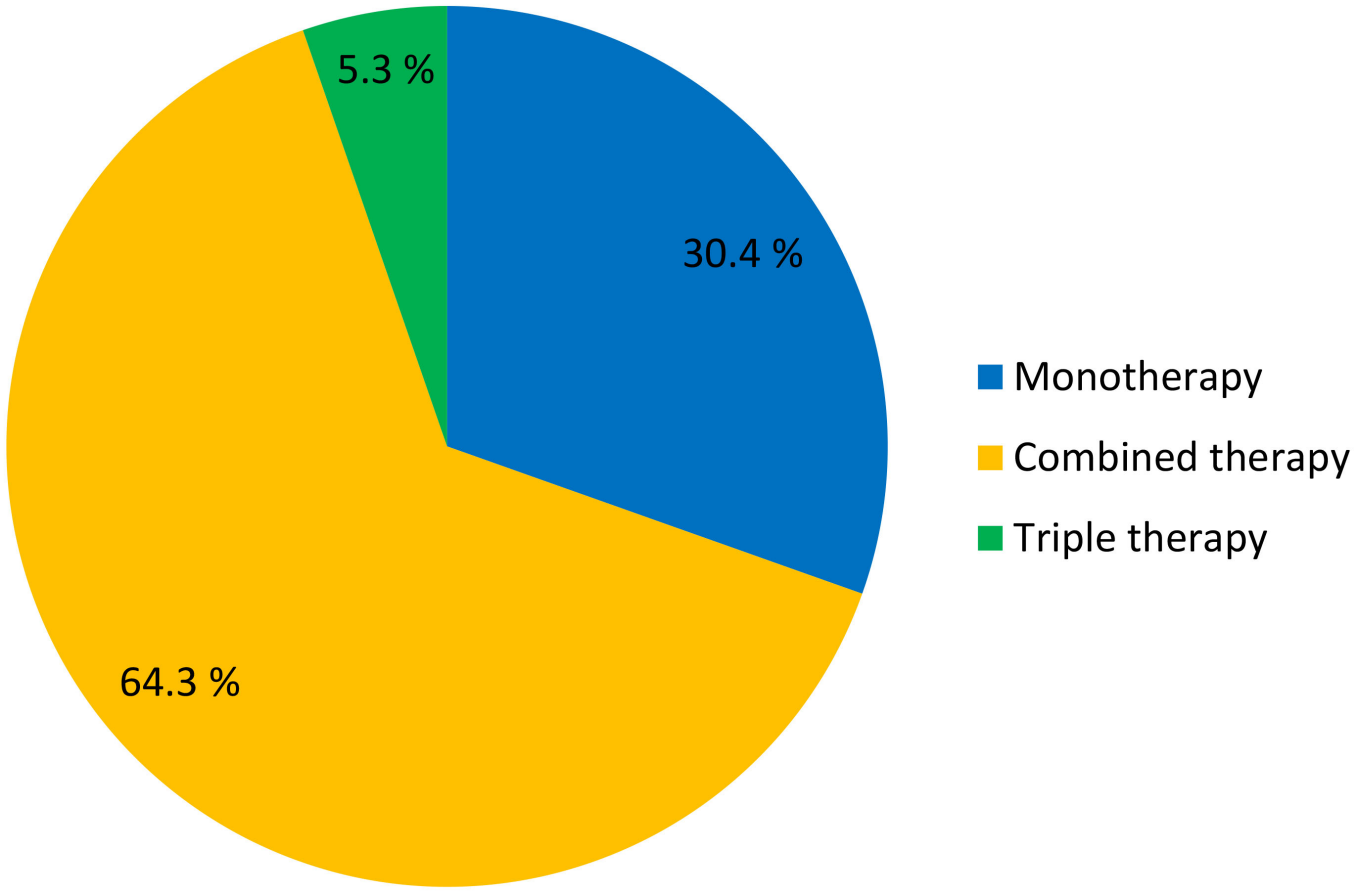
Types of anti-diabetic therapy among patients with type 2 diabetes mellitus (n=910).

Treatment of diabetes mellitus involved using from one to three combined drugs. Monotherapy was prescribed for 277 (34.4%) patients, combined therapy was prescribed for 585 (64.3%) patients, and triple therapy was prescribed for 48 (5.3%) patients (**Figure 2**). The most common hypoglycemic medications used in treatment of patients with type 2 diabetes was a combination of metformin with sulfonylureas which was prescribed for 533 (58.6%) patients. Other less common medications were metformin monotherapy in 112 (12.3%) patients and sulfonylurea monotherapy in 84 (9.2%) patients. Insulin monotherapy was prescribed for 109 (12%) patients, while insulin added to one or more oral hypoglycemic drugs was prescribed for 55 (6%) patients. Most hypoglycemic drugs failed to achieve adequate glycemic control in patients with type 2 diabetes. Further details about pharmacotherapy patterns of type 2 diabetes are enlisted in **Table 5**.

**Table 5:** Prescribing patterns of anti-diabetic drugs among type 2 diabetes mellitus patients (n=910).

Hypoglycemic drugs	Total patients	Glycemic control	
		Adequate	Inadequate
	n (%)	n (%)	n (%)
Metformin + sulfonylureas	533 (58.6)	46 (8.6)	487 (91.4)
Metformin	112 (12.3)	22 (19.6)	90 (80.4)
Insulin	109 (12)	22 (20.2)	87 (79.8)
Sulfonylureas	84 (9.2)	17 (20.2)	67 (79.8)
Metformin + insulin	30 (3.1)	2 (6.7)	28 (93.3)
Metformin + sulfonylureas + insulin	21 (2.3)	1 (4.8)	20 (95.2)
Metformin + two sulfonylureas	20 (2.2)	1 (5)	19 (95)
Sulfonylureas + meglitinides	3 (0.3)	1 (33.3)	2 (66.7)
SGLT2 inhibitors	1 (0.1)	1 (100)	0
Metformin + pioglitazone	12 (1.3)	0	12 (100)
Metformin + sulfonylureas + meglitinides	4 (0.4)	0	4 (100)
Meglitinides	2 (0.2)	0	2 (100)
Metformin + meglitinides	2 (0.2)	0	2 (100)
Two sulfonylurea drugs	2 (0.2)	0	2 (100)
Sulfonylureas + insulin	2 (0.2)	0	2 (100)
Metformin + pioglitazone + insulin	2 (0.2)	0	2 (100)
Metformin + SGLT2 inhibitors	1 (0.1)	0	1 (100)
Metformin + pioglitazone + sulfonylureas	1 (0.1)	0	1 (100)
**Total**	910 (100)	109 (12)	801 (88)

SGLT2, sodium glucose co-transporter 2.

Further analysis of data showed that 368 diabetic patients (40.4%) had not received treatment for the comorbid dyslipidemia. Though, adequate dyslipidemia control was found in 22 (6%) of those untreated patients (**Table 6**). These findings could be explained by the effects of anti-diabetic therapy to lower plasma lipids. Other patients were either treated with moderate-intensity statins (46.4%) or high-intensity statins (7.5%). Thus, these results showed that most patients with type 2 diabetes received no or insufficient treatment for the comorbid dyslipidemia. This contravene with the international guidelines which recommend a high-statin therapy for most patients with type 2 diabetes, and may explain the inadequate dyslipidemia control in patients with type 2 diabetes.

**Table 6:** Effects of hypolipidemic and hypoglycemic therapy on dyslipidemia control in patients with type 2 diabetes.

Therapy	Total patients	Dyslipidemia control	
		Adequate	Inadequate
	n (%)	n (%)	n (%)
Dyslipidemia therapy			
No dyslipidemia drug	368 (40.4)	22 (6)	346 (94)
High-intensity statins	68 (7.5)	30 (44.1)	38 (55.9)
Moderate-intensity statins	422 (46.4)	102 (24.2)	320 (75.8)
Moderate-intensity statins + ezetimibe or fibrates or nicotinic acid	43 (4.7)	25 (58.1)	18 (41.9)
Low intensity statins	4 (0.4)	0	4 (100)
Fibrates	5 (0.6)	0	5 (100)
Diabetes mellitus therapy			
Monotherapy	277 (30.4)	55 (19.9)	222 (80.1)
Combined therapy	585 (64.3)	111 (19)	474 (81)
Triple therapy	48 (5.3)	13 (27.1)	35 (72.9)
**Total**	910 (100)	179 (19.7)	731 (80.3)

Moreover, about 19.8% of diabetic patients with comorbid cardiovascular diseases received no pharmacotherapy, 24% were treated with one drug, 33.5% were treated with combined therapy, and 17.9% were treated with triple therapy. These findings may explain the inadequate control of hypertension observed in most patients with type 2 diabetes. The pharmacotherapy patterns used in treatment of the comorbid cardiovascular diseases in patients with type 2 diabetes mellitus are presented in **Table 7**.

**Table 7:** Treatment patterns of the cardiovascular comorbidities in patients with type 2 diabetes (n=910).

Treatment	Total patients n (%)
**Comorbid cardiovascular diseases**	839 (92.2)
No cardiovascular drugs	166 (19.8)
Treated with one drug	201 (24)
Treated with two drugs	281 (33.5)
Treated with three drugs	150 (17.9)
Treated with four drugs	36 (4.2)
Treated with five drugs	5 (0.6)

## Discussion

The present study screened and assessed metabolic syndrome among patients with type 2 diabetes mellitus and the correlation with glycemic control. Using the NCEP ATP III criteria, metabolic syndrome was identified in 87.5% of type 2 diabetes patients. These results are higher than another study that found the prevalence of metabolic syndrome to be up to 34.2% in the US population (8), as well as studies conducted on Indian, Egyptian, and Ethiopian type 2 diabetes mellitus populations (65%, 55%, and 53.2% respectively) (9–11). On the other hand, the results agree with studies conducted on Saudi type 2 diabetes patients, which found that 85.8% have metabolic syndrome (12). In concordance with previous reported results, the prevalence pattern of metabolic syndrome increased with age (13); this could be attributed to evolution of insulin resistance, increased adipose tissue, and hormonal alterations. A higher prevalence of metabolic syndrome was found in female patients as compared to male patients, as similarly reported in previous studies (14).

In the present study, insulin resistance was identified in all patients with type 2 diabetes, which agrees with previous literature. Insulin resistance is a primary precursor for development of diabetes mellitus type 2 and is thought to be a primary denominator underlying the pathophysiology of metabolic syndrome (15). Patients with metabolic syndrome have slightly elevated fasting blood sugar in the pre-diabetic range of 100−125 mg/dL or a post-prandial glucose level of 140–199 mg/dL or greater. Greater amounts of insulin are secreted from pancreatic β cells as a compensatory mechanism to maintain euglycemia in these states. Decompensation eventually occurs and insulin levels are decreased, leading to hyperglycemia and type 2 diabetes mellitus (16–18). A likely explanation is that prolonged high levels of insulin induce down-regulation and desensitization of insulin receptors, leading to insulin resistance (19, 20).

The overall prevalence of abdominal obesity in type 2 diabetes mellitus patients was 62.5% in the present study. Abdominal obesity is associated with the development of enlarged and dysfunctional adipose cells (21), which secrete adipokines like adiponectin and inflammatory cytokines, including interleukins and tumor necrosis factor alpha, which contribute to insulin resistance as well as the pro-inflammatory and pro-hypertensive states associated with visceral obesity (22–24). Lipolysis from visceral fat deposits increases free fatty acid supply to the liver, leading to increased hepatic synthesis of very low density lipoproteins and thus, raised levels of triglycerides (20, 25). Free fatty acids can also accumulate in the pancreas as well as other organs, leading to organ dysfunction, a process known as lipotoxicity (26). Different signaling pathways are involved in possible mechanisms for how lipotoxicity induces insulin resistance in nonadipose tissues. For example, lipid accumulation in skeletal muscles is linked with impaired activation of phosphatidylinositol-3 kinase (PI3K), which activates downstream protein kinase B (Akt) that ultimately phosphorylates and inactivates glycogen synthase kinase 3, promoting glucose storage as glycogen (27, 28). Thus, central obesity could play an essential role in the development of insulin resistance that culminated in having type 2 diabetes mellitus. In particular, central obesity was much more prevalent among female patients (84%) than male patients (42.6%). This finding could be attributed to the sedentary lifestyle and less frequent involvement of women in aerobic exercises in the ultraconservative community in Yemen.

Dyslipidemia is a hallmark of metabolic syndrome. The present study identified decreased levels of HDLs in 95.3%, as well as increased levels of triglycerides among 80.1%, of type 2 diabetes mellitus patients. Overproduction of very low-density lipoproteins seems to be the most important etiology of increased plasma triglycerides in patients with insulin resistance and type 2 diabetes (29). Additionally, those with insulin resistance and type 2 diabetes mellitus have low plasma HDL levels and elevated triglyceride levels (30). The inverse relationship between plasma triglyceride and HDL levels among insulin resistance patients could be explained by the exchange of triglycerides in the very low-density lipoproteins for HDL cholesteryl esters, a process mediated by cholesteryl ester transfer protein (31). In addition, normal insulin-mediated lipoprotein lipase activation is diminished in insulin resistance where patients are relatively insulin deficient and particularly in those with poor glycemic control (32, 33). Thus, dyslipidemia and obesity are risk factors that need to be controlled to impede the development of type 2 diabetes mellitus or to regress its complications.

Elevated blood pressure was found among 83.0% of type 2 diabetes mellitus patients in the present study. This is in concordance with previous results, which showed higher than normal blood pressure values and hypertension among 80% of individuals with metabolic syndrome (34). Several mechanisms have been postulated to explain blood pressure elevation in patients with metabolic syndrome. For example, impaired activation of the PI3K pathway in the case of insulin resistance reduces the antiatherogenic effects of insulin (35, 36). Furthermore, impaired activation of Akt by PI3K results in reduced endothelial nitric oxide synthase activity and production of nitric oxide that mediates smooth muscle relaxation in the blood vessels (37). Additionally, angiotensin I receptors are upregulated by insulin hypersecretion through post-transcriptional mechanisms involving stabilization of receptor mRNA (38). Moreover, the enhanced sympathetic activity stimulates the renin-angiotensin-aldosterone system, leading to renal sodium reabsorption and volume expansion, which further elevates both diastolic and systolic blood pressure.

Most (52.6%) type 2 diabetes mellitus patients enrolled in the present study were on combined therapy, mostly a combination of metformin and sulfonylurea drugs (45.2%). Monotherapy was prescribed for 39.9%, while triple therapy was prescribed for 7.5% of the patients. The tendency to use combination therapy reflects the necessity of intensive control of blood glucose, which is in line with previous findings from Nigeria (39). Adequate glycemic control was only achieved among 15.6% patients, while 84.4% of the patients had inadequate glycemic control. Comparable findings (14.8%) were reported from Bahrain (40), while better glycemic control has been reported from UAE (41%) (41), Oman (35%) (42) and Saudi Arabia (32.3%) (43). Glycemic control is far from findings reported in Netherlands (76%) (44), and Norway (62.8%) (45). The inadequate glycemic control observed in the present study could be attributed to insufficient medications and poor patient compliance and reflects the necessity review pharmacotherapy and adherence among type 2 diabetes patients and is correlated with the high incidence of comorbid cardiovascular complications associated with type 2 diabetes.

Dyslipidemia (97.7%) and hypertension (83.0%) were the most frequent comorbidities in patients with type 2 diabetes mellitus. Other comorbid cardiovascular diseases included ischemic heart disease (28.5%) and congestive heart failure (17.3%). These results are in concordance with previous studies that strongly correlated metabolic syndrome with risk of cerebrovascular diseases in type 2 diabetes mellitus patients (46). Blood pressure was adequately controlled only among 34.2% patients with type 2 diabetes, while adequate plasma lipoprotein levels were only achieved among 23.6%. Reduction of blood pressure values below 140mmHg has been associated with decreased cardiovascular morbidity and mortality (47, 48). Thus, our results indicate that metabolic syndrome was associated with inadequate control of blood pressure and dyslipidemia in patients with type 2 diabetes mellitus.

Recent evidence shows mechanistic roles of new biomarkers including a multiprotein complex called nucleotide-binding domain and leucine-rich repeat protein 3 (NLRP3) inflammasome in the pathogenesis of metabolic syndrome (49). NLRP3 inflammasome regulates production of several inflammatory mediators by including IL-18, and IL-1β which mediates generation of other inflammatory mediators including TNF-α and IL-6 by binding to the IL-1 receptor (50). Thus, self-amplifying cytokine vicious cycle is initiated that promotes progression of insulin resistance, atherosclerosis, and metabolic syndrome (51).

Limitations of the present study include it was observational cross sectional study, and causal relationship cannot be well established on these studies. Implementing therapeutic and lifestyle interventions were also beyond the scope of the study. A prospective cohort study is warranted to confirm the study findings by modulating the risk factors of metabolic syndrome and determining a causal relationship between metabolic syndrome and pharmacotherapy outcomes. Moreover, the role of recent biomarkers including NLRP3 inflammasome and its downstream cytokines including IL-1β in the pathogenesis of metabolic syndrome as well as its comorbid diseases are warranted.

## Conclusions

Metabolic syndrome was highly prevalent among type 2 diabetes mellitus patients. The majority of the patients had either 5 or 4 risk factors of metabolic syndrome while limited number patients had 2 risk factors and no patient displayed a single risk factor of metabolic syndrome. After insulin resistance which was found in all patients, the most common component of metabolic syndrome among patients with type 2 diabetes mellitus was HDL dyslipidemia (94.3%), followed by elevated blood pressure (83%) and triglyceride dyslipidemia (80.1%). Abdominal obesity was predominantly observed among women (84%) than men (42.6%) with type 2 diabetes mellitus. This could be attributed to sedentary life style adopted by most females in Yemen. Metabolic syndrome was associated with inadequate glycemic control as well as suboptimal control of blood pressure and dyslipidemia. Early interventions to treat metabolic syndrome could improve treatment outcomes of diabetes mellitus and prevent the likelihood cardiovascular complications. Moreover, the study reflects an urgent need to review pharmacotherapy, patient counseling, and health education for patients.

## Data availability statement

The original contributions presented in the study are included in the article/supplementary material. Further inquiries can be directed to the corresponding author.

## Ethics statement

The studies involving humans were approved by Institutional Review Board of Wahda University Hospital, Faculty of Medicine, Thamar University. The studies were conducted in accordance with the local legislation and institutional requirements. The participants provided their written informed consent to participate in this study.

## Author contributions

SA: Conceptualization, Data curation, Formal analysis, Funding acquisition, Investigation, Methodology, Project administration, Resources, Software, Supervision, Validation, Visualization, Writing – original draft, Writing – review & editing. MA-D: Conceptualization, Data curation, Formal analysis, Funding acquisition, Investigation, Methodology, Project administration, Resources, Software, Supervision, Validation, Visualization, Writing – original draft, Writing – review & editing. SA-S: Conceptualization, Data curation, Formal analysis, Funding acquisition, Investigation, Methodology, Project administration, Resources, Software, Supervision, Validation, Visualization, Writing – original draft, Writing – review & editing.
